# The earthquake's effect on the men's sexual function; 1 year after the earthquake's occurrence: A cross‐sectional study

**DOI:** 10.1002/hsr2.945

**Published:** 2022-11-24

**Authors:** Abbasali Ebrahimian, Hossein Babaei

**Affiliations:** ^1^ Health in Emergencies and Disasters Group, Faculty of Paramedical Qom University of Medical Sciences Qom Iran; ^2^ Nursing Care Research Center Semnan University of Medical Sciences Semnan Iran; ^3^ Student Research Committee, Nursing School Semnan University of Medical Sciences Semnan Iran

**Keywords:** earthquake, men, psychological disorders, sexual function

## Abstract

**Background and Aims:**

Sexual dysfunctions are one of the health problems after natural disasters that are usually less attention. The purpose of this study was to evaluate the effect of the earthquake on men's sexual functions 1 year after the earthquake.

**Methods:**

This study was a descriptive‐analytical cross‐section study that took place a year after the Kermanshah earthquake. The population studied was all men living in the Kermanshah earthquake. Demographic, socioeconomic, psychological, health situations, facilities availability, and environmental situations after the earthquake and International Index of Erectile Function (IIEF) were randomly distributed among men affected by the earthquake. Participants returned the questionnaires through the mail. Based on the IIEF cut‐point score, men were divided into two groups: those with sexual dysfunction (Group A) and without sexual dysfunction (Group B).

**Results:**

In this study, 225 married men participated. The prevalence of sexual dysfunction in earthquake‐affected men was 44.9%. The mean total IIEF scores in the A and B groups were 43.47 ± 7.82 and 62.11 ± 6.39, respectively. There was a significant difference between the total and all subcategories IIEF scores in the two groups (*p* < 0.001). There was a statistically significant difference between the age (*p* < 0.001), child numbers (*p* < 0.017), current live location (*p* < 0.001), social support after the earthquake (*p* = 0.033), underlying disease (*p* < 0.001), availability of sanitary toilets (*p* < 0.001) and bathrooms (*p* = 0.002), and total IIEF scores between the two groups (*p* < 0.001).

**Conclusions:**

Approximately half of the earthquake‐affected men had sexual dysfunctions. The men's age, child numbers, current live location, social support, underlying disease, and availability of sanitary toilets and bathrooms were influential in the severity of men's sexual dysfunctions after the earthquake. Therefore, crisis managers, policymakers, psychiatrists, and psychologists should pay enough attention to men's sexual dysfunction after earthquakes.

## 1 INTRODUCTION

Iran is one of the most earthquake‐stricken countries in the world, and many earthquakes have been recorded.^1^ The Kermanshah earthquake occurred at 21:48:16 on Sunday, November 12, 2017, near Ozgole, Kermanshah province (near the Iran‐Iraq border), 32 km southwest of the city of Halbacheh (Iraq). The power of this earthquake was 7.3 km on the Richter torque scale, and its center was 5 km away from the city of Ozgole in Kermanshah province. The quake was felt in southeast Turkey, Kuwait, and northern parts of Saudi Arabia. This earthquake affected 150,000 people in Kermanshah province. According to official reports, 630 were dead, and 9388 were injured. Besides, about 70,000 lost their homes.^2^, ^3^

Sexual disorders and fertility health are the most common problems after natural disasters such as earthquakes.^4^ High stillbirth rates, decrease in live birth rate, general marriage fertility rate, contraceptive methods coverage, sexual violence, and prevalence of sexually transmitted diseases are among the essential consequences of earthquakes concerning sexual and fertility health.^5^, ^6^, ^7^ However, it is an often neglected issue during natural disasters.

Having healthy sexual relations requires optimal physical and mental health, and sexual intercourse should be at the right time. Usually, after severe emotional shocks such as stress, anxiety, and depression, sexual relations are not as good as before.^8^ Biophysical factors, physical problems and diseases, spinal traumatic stress disorders, and posttraumatic stress disorder also affect the sexual health of individuals,^9^, ^10^, ^11^, ^12^, ^13^ which all increase after an earthquake.

Paying inadequate attention to the sexual health status of individuals affected by earthquakes may cause a phenomenon called “rape.” Bartels,^14^ in a study on individuals who experienced postearthquake rape in South Kivu Province and Panzi Hospital, reported that between 2004 and 2008, about 4311 cases of rape were referred to the hospital, and most of them were referred 10.4 months after the occurrence of rape. Also, Kung and Gell,^15^ in a report on raping women and girl's 1‐year after the Haiti earthquake, described the statistics as shocking. Also, no precise information is provided. On the other hand, sexual disorders in men can cause sexual disorders in their partners and may result in disturbances in their relations.^16^ Therefore, natural disasters and related problems can cause or exacerbate psychological and sexual issues. Because few studies were conducted on postdisaster sexual problems in Iran and the world, the current study was performed. This study's objective was to evaluate the earthquake's effect on the men's sexual function 1 year after the Kermanshah earthquake.

## 2 METHODS

### 2.1 Study design and participants

This descriptive‐analytical cross‐sectional study collected information about the sexual health of the earthquake‐affected men in the year ending November 13, 2018. The study population was all men living in Sar‐e‐Pol‐e‐Zahab and Kermanshah cities when the Kermanshah earthquake occurred and had the inclusion criteria.

Inclusion criteria:

1. Reside in the earthquake area from the time of the earthquake until the completion of the questionnaire.
2. The participant has expressed willingness to participate in the research and completed the informed consent form.
3. Be alert and fully able to answer the questionnaire's questions.
4. There should be no pain or acute problems when completing the questionnaire.
5. The participants be married and should not have any restrictions on the sexual activity before and after the earthquake.
6. No history of previously known physical and mental illnesses.

Exclusion criteria:

1. Unwillingness to continue participating in the study.
2. Failure to complete and send the questionnaire.

### 2.2 Sample size

The sample size was estimated based on a preliminary study conducted on 20 earthquake‐affected men with a mean and standard deviation International Index of Erectile Function score of 52.80 ± 13.53.

The sample size was computed using the G^*^power software with a confidence interval of 95%, a test power of 99%, and an effect size of 0.150. The minimal sample size was obtained as 212 patients per group.

### 2.3 Measures

Data collection tools were demographic, socioeconomic, psychological, health situations, facilities availability, and environmental situations after the earthquake questionnaire and International Erectile Performance Index‐15 (IIEF‐15). After receiving informed consent, the participant completed the patients' demographic information and IIEF‐15 questionnaire. This information includes age, level of education, number of children, duration of the marriage, type of job, socioeconomic status, psychological status, health situation, facilities availability, and environmental situations after the earthquake.

The IIEF‐15 questionnaire was used to determine the past year's quality of the sexual function in the participants. This present study utilized the 15‐question format of this questionnaire. This questionnaire is a brief, reliable, validated, multidimensional, self‐administered investigation that has helped assess erectile dysfunction. IIEF‐15 evaluates the presentation of male sexual dysfunction. The minimum score of this questionnaire was 5, which was interpreted as a lack of sexual quality functions and the maximum score was 75, demonstrating the highest amount of sexual quality functions (Table 1).^17^ This study evaluated the five components of sexual function: erectile function, orgasmic function, sexual desire, intercourse satisfaction, and overall satisfaction from sexual activity. Erectile function was evaluated based on six questions, yielding a maximum score of 30 points.

**Table 1:** IIEF domain scoring

Domain	Domain scoring				
	Items	Score range	Minimum score	Maximum score	Abnormal results
Erectile function	1, 2, 3, 4, 5, 15	0 (or 1)–5	1	30	<25 points
Orgasm function	9, 10	0–5	0	10	<9 points
Sexual desire	11, 12	1–5	2	10	<9 points
Intercourse satisfaction	6, 7, 8	0–5	0	15	<13 points
Overall satisfaction	13, 14	1–5	2	10	<9 points

Abbreviation: IIEF, Index of Erectile Function.

Results below 25 points were considered abnormal. The orgasmic function was evaluated using two questions, yielding a maximum score of 10 points. Results below 9 points were considered abnormal. Sexual desire was also evaluated using two questions, yielding a maximum score of 10 points, and results below 9 points were considered abnormal. Intercourse satisfaction was evaluated based on three questions, yielding a maximum score of 15 points, and the threshold value was 13 points. Overall satisfaction from sexual activity was evaluated using two questions, yielding a maximum score of 10 points, and results below 9 points were considered abnormal (Table 1).^18^ In the present study, a score of 53 was considered the cut point for total IIEF scores.^19^ This questionnaire has validity for erictical dysfunction and has been used in some studies.^20^, ^21^ Rosen et al.^17^ confirmed the reliability of the IIEF by calculating a Cronbach's *α* of 0.91. A Cronbach's *α* of 0.94 also confirmed the reliability of the IIEF tool in the present study.

### 2.4 Procedures

Participants were randomly selected from the displaced and nondisplaced population following the 2017 Kermanshah earthquake. The researcher divided the earthquake‐stricken area of Sarpol‐e Zahab from the map into four parts. Then, he randomly went to the participants' houses, Conex, and Tents.

After explaining the objectives and methodology of the study, written informed consent was obtained from participants. All participants were given a stamped envelope containing questionnaires with an address to send back the questionnaire through the national post service. In each region, 75 questionnaires and, in total, 300 questionnaires were distributed. Of these, 263 questionnaires were filled and sent back. Thirty‐eight questionnaires were incomplete and were excluded from the study, and analyzed the data related to 225 questionnaires. After sampling, the data were entered into SPSS‐16 software, and the data were analyzed. The samples were divided into two groups with sexual dysfunction (Group A) and without sexual dysfunction (Group B) based on the cut‐point score of 53 on the IIEF questionnaire. Then, the relationship between demographic, socioeconomic, psychological, health situations, facilities availability, and environmental situations after the earthquake variables in people with and without sexual dysfunction were analyzed.

### 2.5 Statistical methods

All analyses were performed using SPSS‐16 software (SPSS Inc). The Kolmogorov–Smirnov test showed that the data distribution was normal. The data were analyzed using descriptive statistics, including frequency, mean, and standard deviation. The Independent sample *t*‐test demonstrated the relationship between the participant's age, BMI, number of children, ILEF scores, and IIEF subcategories in the A and B groups. The Mann–Whitney *U*‐test showed the relationship between the demographic, socio‐economical, psychological, health situation, facility availability, and environmental situations in the A and B groups. The ANOVA test showed the correlation between the socioeconomical, psychological, health situation, facility availability, and environmental situations and the mean of IIEF scores within the A and B groups. The level of statistical significance was set at *p* < 0.05 for all statistical analyses.

### 2.6 Ethical considerations

The Ethics Committee of Semnan University of Medical Sciences approved this study protocol (approval code: IR.SEMUMS.REC.1397.214). We coordinated with the Kermanshah University of Medical Sciences before sampling. The study objectives and procedure were also explained to the participants, and informed consent was obtained.

## 3 RESULTS

Three hundred questionnaires were distributed among those with the inclusion criteria, and 263 questionnaires were returned. Thirty‐eight questionnaires were excluded from the study due to incompleteness, and data from 225 questionnaires were analyzed. At first, the participants in the study were divided into two groups, with (A group) and without sexual dysfunction groups (B group), based on the total score cut‐point of IIEF. 101 (44.9%) participants were in the group with sexual dysfunction, and 124 (55.1%) were in the group without sexual dysfunction.

In the with sexual dysfunction group, the participants' mean age, BMI, and child number were 44.73 ± 8.79, 27.29 ± 4.34, and 3.02 ± 1.36, respectively. In the without sexual dysfunction group, the participants' mean age, BMI, and child number were 38.25 ± 8.29, 26.25 ± 3.13, and 2.63 ± 1.10, respectively (Table 2).

**Table 2:** Comparison between male demographic data in the with and without sexual dysfunction groups and the effect of these variables on IIEF scores

	Groups	With sexual dysfunction		Without sexual dysfunction		*p* Value
Variables		*n* (%)	IIEF Mean ± SD	*n* (%)	IIEF Mean ± SD	*n* (%)
Age	Young (18–35 years)	14 (13.9)	44.64 ± 5.31	49 (39.5)	63.44 ± 7.24	<0.001^a^
	Middle‐aged (36–45 years)	37 (36.6)	45.62 ± 6.55	47 (37.9)	60.85 ± 5.42	
	Adult (46–65 years)	50 (49.5)	41.56 ± 8.85	28 (22.6)	61.89 ± 6.05	
	Total mean ± SD	44.73 ± 8.79	—	38.25 ± 8.29	—	
BMI	Under‐weight (<18.5)	2 (2)	43.00 ± 2.82	0 (0)	—	0.137^a^
	Normal (18.5–24.9)	30 (29.7)	44.10 ± 8.97	45 (36.3)	62.33 ± 6.89	
	Over‐weight (25–29.9)	49 (48.5)	44.36 ± 6.40	68 (54.8)	62.14 ± 6.11	
	Obese (>30)	20 (19.8)	44.40 ± 9.13	11 (8.9)	61.00 ± 6.44	
	Total mean ± SD	27.29 ± 4.34	—	26.25 ± 3.13	—	
Educational status	Under‐diploma	31 (30.7)	40.74 ± 9.11	39 (31.5)		0.973^a^
	Diploma	23 (22.8)	44.73 ± 7.62	27 (21.8)		
	College education	47 (46.5)	44.65 ± 6.63	58 (46.8)		
Child's number	0	4 (4)	37.50 ± 4.04	5 (4)	62.20 ± 8.72	0.017^a^
	1	10 (9.9)	47.80 ± 3.88	14 (11.3)	64.50 ± 6.74	
	2	20 (19.8)	44.85 ± 4.60	31 (25)	64.12 ± 6.83	
	3	26 (25.7)	43.96 ± 9.24	50 (40.3)	60.42 ± 5.68	
	4	33 (32.7)	42.81 ± 7.87	20 (16.1)	62.20 ± 6.16	
	5	3 (3)	48.66 ± 1.15	4 (3.2)	58.75 ± 3.50	
	6	5 (5)	32.80 ± 9.73	0 (0)	—	
	Total mean ± SD	3.02 ± 1.36	—	2.63 ± 1.10	—	
Current live location	Permanent housing	57 (56.4)	44.38 ± 7.80	107 (86.3)	61.51 ± 6.08	<0.001^a^
	Connex container	38 (37.6)	41.81 ± 8.00	13 (10.5)	65.84 ± 7.78	
	Tent	6 (5.9)	45.33 ± 6.85	4 (3.2)	66.00 ± 5.77	
Types of Job	Government's employee	49 (48.5)	45.36 ± 5.99	47 (37.9)	62.14 ± 5.90	0.311^a^
	Worker	9 (8.9)	43.11 ± 4.13	15 (12.1)	61.20 ± 7.62	
	Farmer	4 (4)	43.50 ± 1.00	15 (12.1)	57.60 ± 2.35	
	Rancher	11 (10.9)	36.45 ± 9.49	8 (6.5)	63.87 ± 6.46	
	Driver	6 (5.9)	39.00 ± 9.20	13 (10.5)	65.46 ± 6.46	
	Unemployed	2 (1.6)	41.00 ± 2.82	2 (1.6)	60.50 ± 4.94	
	Others jobs	20 (20.2)	44.15 ± 10.63	24 (19.3)	55.33 ± 1.15	

Abbreviations: IIEF, Index of Erectile Function; n, number, SD, standard deviation.

a Based on Mann–Whitney *U*‐test.

In this study, 164 (72.9%) participants at the sampling time (1 year after the earthquake) lived in permanent housing, and the rest resided in Connex containers and tents. Also, 96 (42.7%) participants were government employees, and others worked in other occupations. 105 (46.7%) participants also had a university education (Table 2).

Mann–Whitney *U*‐test showed a significant association between age (*p* < 0.001), child number (*p* = 0.017), and current live location (*p* < 0.001) among the A and B groups. However, there was no significant difference between the BMI, educational status, and types of jobs after the earthquake among the A and B groups (*p* > 0.05) (Table 2).

The Pearson's correlation coefficient test showed a negative correlation between age, MMI, and the number of children with IIEF scores. In this way, with increasing age, BMI, and the number of children, the IIEF score decreased. These findings mean that with increasing age, BMI, and the number of children, the severity of sexual disorders in earthquake victims increased.

All participants in the with sexual dysfunction group in all subgroups of IIEF tools (Erectile function, Orgasmic function, Sexual desire, Intercourse satisfaction, and Overall satisfaction) had reduced function. In the with sexual dysfunction group, sexual dysfunction prevalence in the IIEF tools subcategories for Erectile function, Orgasmic function, Sexual desire, Intercourse satisfaction, and Overall satisfaction was 48.4%, 72.6%, 91.1%, 83.1%, and 76.6%, respectively. Independent samples *t*‐test showed a significant difference between Erectile function, Orgasmic function, Sexual desire, Intercourse satisfaction, and Overall satisfaction subscales in groups A and B (*p* < 0.001). The mean total IIEF scores in the A and B groups were 43.47 ± 7.82 and 62.11 ± 6.39, respectively. There was a significant difference between total IIEF scores in the two groups (*p* = 0.000) (Table 3).

**Table 3:** The male sexual functions after the earthquake in with and without sexual dysfunction groups based on IIEF subscales

Groups		With sexual dysfunction		Without sexual dysfunction		*p* Value^a^
IIEF subscales		*N* (%)	Mean ± SD	*N* (%)	Mean ± SD	
Erectile function	Normal function	0 (0)	17.71 ± 3.50	64 (51.6)	25.46 ± 3.09	<0.001
	Decreased function	101 (100)		60 (48.4)		
Orgasmic function	Normal function	0 (0)	6.00 ± 1.52	34 (27.4)	8.64 ± 1.06	<0.001
	Decreased function	101 (100)		90 (72.6)		
Sexual desire	Normal function	0 (0)	5.47 ± 1.62	11 (8.9)	8.00 ± 1.11	<0.001
	Decreased function	101 (100)		113 (91.1)		
Intercourse satisfaction	Normal function	0(0)	7.78 ± 1.79	21 (16.9)	11.66 ± 1.86	<0.001
	Decreased function	101 (100)		103 (83.1)		
Overall satisfaction	Normal function	0 (0)	6.49 ± 1.61	29 (23.4)	8.33 ± 1.33	<0.001
	Decreased function	101 (100)		95 (76.6)		
Total IIEF	Normal function	0 (0)	43.47 ± 7.82	124 (100)	62.11 ± 6.39	<0.001
	Decreased function	101 (100)		0 (0)		

Abbreviation: IIEF, Index of Erectile Function.

a Based on independent sample *t*‐test.

After the earthquake, 12 (11.9%) participants in the A group and 5(4%) participants in the B group did not receive any social support. In the A group, 11 (10.9%) participants were not affected by the economic problems caused by the earthquake, and 26 (25.7%) participants were highly affected by the economic problems caused by the earthquake. After the earthquake, 97 (96%) participants in the A group and 121 (97.6%) participants in the B group did not receive psychological consultations. In the A group, 106 (85.5%) participants did not experience any physical injury during the earthquake. 40 (39.6%) participants in the A group and 20 (16.1%) participants in the B group had underlying diseases. In the A group, 12 (11.9%) and in the B group, 15 (12.1%) participants had enough sleep, but the rest of the participants in both groups experienced some sleep disturbance after the earthquake. 55 (54.5%) participants in the A group and 96 (77.4%) in the B group had access to appropriate food more often or always. The A group, 26 (25.7%), and the B group, 31 (25%), always had access to appropriate and sufficient fruits and vegetables. Mann–Whitney *U*‐test showed a significant association between social support after the earthquake and underlying disease among the A and B groups (*p* < 0.001). However, there was no significant difference between the economic losses from the earthquake, use of psychological counseling after the earthquake, enough sleep, physical injuries caused by earthquakes, and acceptable use of appropriate foods and suitable fruits after the earthquake among the A and B groups (*p* > 0.05) (Table 4).

**Table 4:** Comparison of socioeconomic, psychological, and health situations in the with and without sexual dysfunction groups and the effect of these variables on IIEF scores

Groups		With sexual dysfunction		Without sexual dysfunction		*p* Value^a^
Variables		*n* (%)	IIEF Mean ± SD	*n* (%)	IIEF Mean ± SD	
Social support after the earthquake	Very high	24 (23.8)	44.70 ± 5.43	33 (26.6)	64.60 ± 6.49	0.033
	High	23 (22.8)	43.73 ± 8.98	47 (37.9)	61.17 ± 6.16	
	Moderate	36 (35.6)	44.22 ± 7.00	30 (24.2)	61.36 ± 6.37	
	Low	6 (5.9)	48.50 ± 2.34	9 (7.3)	60.33 ± 5.87	
	None	12 (11.9)	35.75 ± 9.55	5 (4)	62.20 ± 6.90	
	*p* Value^b^	0.003		0.127		—
Economic losses from the earthquake	Very high	26 (25.7)	41.57 ± 10.29	33 (26.6)	60.78 ± 6.22	0.248
	High	36 (35.6)	46.55 ± 4.16	27 (21.8)	62.03 ± 6.62	
	Moderate	16 (15.8)	42.93 ± 5.53	33 (26.6)	64.24 ± 6.06	
	Low	12 (11.9)	40.08 ± 9.61	7 (5.6)	59.57 ± 6.52	
	None	11 (10.9)	42.36 ± 8.98	24 (19.4)	61.83 ± 6.47	
	*p* Value^b^	0.043		0.182		
Use psychological counseling after the earthquake	Yes	4 (4)	36.70 ± 10.75	3 (2.4)	58.87 ± 2.79	0.344
	No	97 (96)	44.21 ± 7.13	121 (97.6)	62.33 ± 6.51	
	*p* Value^b^	0.003		0.139		
Earthquake bodily injury	Yes	18 (14.5)	44.84 ± 6.49	25 (24.8)	60.61 ± 5.99	0.053
	No	106 (85.5)	43.02 ± 8.20	76 (75.2)	62.36 ± 6.45	
	*p* Value^b^	0.317		0.283		
Underlying disease	Yes	40 (39.6)	40.05 ± 8.93	20 (16.1)	57.95 ± 3.34	<0.001
	No	61 (60.4)	45.72 ± 6.10	104 (83.9)	62.91 ± 6.54	
	*p* Value^b^	0.000		0.001		
Enough sleep after the earthquake	Always	12 (11.9)	45.75 ± 1.86	15 (12.1)	57.33 ± 4.28	0.219
	Often	43 (42.6)	45.30 ± 7.69	58 (46.8)	61.98 ± 6.35	
	Sometimes	20 (19.8)	37.45 ± 7.17	35 (28.2)	62.02 ± 6.36	
	Rarely	24 (23.8)	44.66 ± 7.74	16 (12.9)	67.25 ± 4.69	
	Never	2 (2)	36.50 ± 13.43	0 (0)	—	
	*p* Value^b^	0.001		0.000		
Adequate and proper nutrition after the earthquake	Always	21 (20.8)	44.23 ± 8.28	45 (36.3)	62.62 ± 6.26	0.051
	Often	54 (53.5)	43.09 ± 6.58	51 (41.1)	61.21 ± 6.63	
	Sometimes	21 (20.8)	43.61 ± 10.19	23 (18.5)	63.34 ± 6.53	
	Rarely	5 (5)	43.80 ± 9.49	4 (3.2)	62.50 ± 3.00	
	Never	0 (0)	—	1 (0.8)	55.00 ± 0.00	
	*p* Value^b^	0.953		0.499		
Sufficient and suitable fruit after the earthquake	Always	26 (25.7)	45.92 ± 5.38	31 (25)	61.77 ± 5.56	0.873
	Often	29 (28.7)	42.68 ± 6.85	43 (34.7)	61.79 ± 6.75	
	Sometimes	30 (29.7)	40.96 ± 9.42	27 (21.8)	62.18 ± 6.62	
	Rarely	13 (12.9)	46.92 ± 6.94	17 (13.7)	62.41 ± 7.81	
	Never	3 (3)	40.00 ± 13.85	6 (4.8)	65.00 ± 1.09	
	*p* Value^b^	0.057		0.839		

Abbreviation: IIEF, Index of Erectile Function.

a Based on Mann–Whitney *U*‐test.

b Based on the ANOVA test.

In the A group, there was a statistically significant difference between the mean scores of IIEF and the level of social support after the earthquake, economic losses from the earthquake, use of psychological counseling after the earthquake, the underlying disease, and enough sleep after the earthquake (*p* < 0.001). In the B group, there was a statistically significant difference between the mean IIEF scores and underlying illness and enough sleep after the earthquake (*p* < 0.001) (Table 4).

In the A group, 66 (65.3%) participants were satisfied with access to electricity, 53 (52.5%) satisfied with access to sanitary water, 34 (33.7%) satisfied with access to a hygienic toilet, 42 (41.6%) satisfied with access to the sanitary bathroom and 25 (24.8%) satisfied with access to environmental cleanliness (Table 5).

**Table 5:** Comparison of facilities availability and environmental situations after the earthquake in the with and without sexual dysfunction groups and the effect of these variables on IIEF scores

Groups		With sexual dysfunction		Without sexual dysfunction		*p* Value^a^
Variables		*n* (%)	IIEF Mean ± SD	*n* (%)	IIEF Mean ± SD	
Availability of electric power	Always	66 (65.3)	43.78 ± 7.37	87 (70.2)	62.88 ± 6.08	0.525
	Often	33 (32.7)	42.87 ± 8.97	32 (25.8)	61.00 ± 7.05	
	Sometimes	2 (2)	43.023 ± 0.45	5 (4)	55.80 ± 1.64	
	Rarely	0 (0)	—	0 (0)	—	
	Never	0 (0)	—	0 (0)	—	
	*p* Value^b^	0.978		0.027		
Availability of sanitary water	Always	53 (52.5)	43.00 ± 8.69	60 (48.4)	63.06 ± 5.81	0.784
	Often	32 (31.7)	44.78 ± 6.08	50 (40.3)	62.10 ± 7.24	
	Sometimes	15 (14.9)	43.46 ± 7.12	8 (6.5)	59.62 ± 4.27	
	Rarely	1 (1)	17.00 ± 0.00	6 (4.8)	56.00 ± 1.09	
	Never	0 (0)	—	0 (0)	—	
	*p* Value^b^	0.151		0.043		
Availability of sanitary toilet	Always	34 (33.7)	44.17 ± 9.33	73 (58.9)	62.02 ± 6.11	<0.001
	Often	47 (46.5)	43.59 ± 6.79	42 (33.9)	61.35 ± 6.60	
	Sometimes	20 (19.8)	42.00 ± 7.51	9 (7.3)	66.33 ± 6.78	
	Rarely	0 (0)	—	0 (0)	—	
	Never	0 (0)	—	0 (0)	—	
	*p* Value^b^	0.715		0.104		
Availability of sanitary bathroom	Always	42 (41.6)	44.57 ± 8.51	76 (61.3)	62.50 ± 6.05	0.002
	Often	46 (45.5)	42.86 ± 7.58	41 (33.1)	62.29 ± 7.08	
	Sometimes	12 (11.9)	43.33 ± 4.53	6 (4.8)	57.16 ± 3.65	
	Rarely	1 (1)	27.00 ± 0.000	1 (0.8)	55.00 ± 0.00	
	Never	0 (0)	0 (0)	0 (0)	—	
	*p* Value^b^	0.198		0.162		
Optimal environmental cleanliness	Always	25 (24.8)	42.84 ± 10.10	33 (26.6)	62.21 ± 6.13	0.578
	Often	33 (32.7)	46.09 ± 4.69	43 (34.7)	63.39 ± 6.88	
	Sometimes	34 (33.7)	41.91 ± 8.22	39 (31.5)	60.79 ± 6.17	
	Rarely	9 (8.9)	41.55 ± 6.98	6 (4.8)	59.00 ± 5.47	
	Never	0 (0)	—	3 (2.4)	66.47 ± 0.23	
	*p* Value^b^	0.122		0.205		

a Based on Mann–Whitney *U*‐test.

b Based on the ANOVA test.

In the B group, 87 (70.2%) participants were satisfied with access to electricity, 60 (48.4%) satisfied with access to sanitary water, 73 (58.9%) satisfied with access to a hygienic toilet, 76 (61.3%) satisfied with access to the sanitary bathroom and 33 (26.6%) satisfied with access to environmental cleanliness. The Mann–Whitney *U*‐test showed a significant association between the availability of hygienic toilets and sanitary bathrooms among the groups with and without sexual dysfunction (*p* < 0.05). In the A group, there was no statistically significant relationship between the scores of IIEF and facilities availability and environmental situations variables (*p* > 0.05). In the B group, there was a statistically significant difference between the mean IIEF scores and the availability of electric power and sanitary water (*p* < 0.05) (Table 5).

## 4 DISCUSSION

This study evaluated the effect of the earthquake on the sexuality function of men affected by the earthquake. The prevalence of sexual dysfunction in earthquake‐affected men was 44.9%. The mean total IIEF scores in the A and B groups were 43.47 ± 7.82 and 62.11 ± 6.39, respectively. There was a significant difference between the total and all subcategories IIEF scores in the two groups (*p* < 0.05). The earthquake‐affected men experienced more severe sexual dysfunction. Researchers have not found any study on sexual dysfunction in men after earthquakes. However, some studies are conducted on the prevalence of sexual dysfunctions in Iran using IIEF. For example, Pasha et al.^22^ showed that the average IIEF in infertile men was 58.30 ± 8.52. Comparing the data of Pasha's study with the present study shows that sexual disorders in earthquake‐affected men who did not have fertility problems were more severe than infertile men. This finding indicates the strong impact of the earthquake on men's sexual health.

One year after the earthquake, all earthquake‐affected men in both groups had problems in at least one of the subcategories of sexual dysfunction. Both groups had a high prevalence of erectile dysfunction, orgasm dysfunction, sexual desire dysfunction, intercourse satisfaction dysfunction, and overall satisfaction. In Pasha et al.^22^ study, most participants had problems with orgasm function and Sexual desire, consistent with some current study results. Also, Lotti and Maggi^23^ also reported sexual disorder and sexual satisfaction as the most common sexual disorder. McCabe et al.^24^ reported erectile disorder and orgasm disorder as the most common sexual disorder. These findings show that sexual dysfunction 1 year after the earthquake is different from other people in society, and their prevalence is significantly higher. Therefore, crisis managers and psychologists should consider sexual dysfunctions after earthquakes.

There was a statistically significant difference between the mean scores of IIEF and the participants' age between the A and B groups. Sexual dysfunction was more severe in older men than in younger men. Moreira et al.^25^ showed in a study that increasing age was a significant predictor of a lack of sexual interest and erectile difficulties in men. Geerkens et al.^26^ in a review study, showed that the severity of sexual disorders slightly increases with age. In the present study, the severity of sexual disorders increased with age. It seems that the increased in life responsibilities, the rise in the number of children, the fatigue caused by trying to help people affected by the earthquake, the inability to provide the vital needs of life after the earthquake, and the inappropriateness of the living environment for sexual activity, have caused an increase in the severity of sexual dysfunction in the earthquake‐affected men. Geerkens and colleagues, in a systematic review study, showed that lack of sexual desire was reported to vary between 12% and 51.6% for men aged ≥60 year, 20% and 65.9% for men aged ≥70 year, and 40% and 82.4% for men aged ≥80 year. The percentages of men bothered by ED were as follows: 14.3%–70% for men aged ≥60 year, 6.7%–48% for men aged ≥70 year, and 38% for men aged ≥80 year. A substantial number of men (50.3%–92%) find a normal sex life vital as they age and remain sexually active. Only a minority of older adults seek help from healthcare professionals or use medication for SD.^26^

There was a statistically significant difference between the mean scores of IIEF and the participants' child's numbers and current live location between the A and B groups. Sexual dysfunction was more severe in the participants with more child's number and people who lived in Connex containers after the earthquake. In a study, Abrams et al.^27^ showed that one factor affecting sexual dysfunction is the number of children under 18‐year‐old. Living with children in a small environment, such as a Tent or a Connex container, prevents couples from performing proper sexual activity. Therefore, it is recommended to consider at least two Tents or Connex containers for married people who have children in disasters.

In the current study, there was no statistically significant relationship between the body mass index, education level, and type of occupation of earthquake‐affected men with the mean of their total IIEF scores between groups A and B. Mykoniatis et al.^28^ and Dursun et al.^29^ showed in a study that body mass index is effective on sexual dysfunctions, and the severity of sexual dysfunction is higher in men who have a higher body mass index. In the present study, the reason for the nonsignificance of the difference between BMI in the two groups is that the participants in the two groups were almost equal in terms of BMI.

Bonde^30^ showed in his study that occupational hazards could affect men's sexual activity and fertility. Yafi et al.^31^ showed in a study that occupation can affect men's sexual dysfunction. However, Omar et al.^32^ showed in a study that being employed has no effect on sexual dysfunction in men with diabetes. The result of the study by Omar and his colleagues confirms the result of the present study and shows that if men suffer from an illness or a crisis such as an earthquake, being employed or not will affect their sexual disorders. This finding means that men are greatly affected by crises and disasters.

It has been shown in many studies that the level of education and especially the level of awareness of people about sexual dysfunctions play a crucial role in reducing sexual dysfunctions.^33^, ^34^ In the present study, two groups were homogeneous regarding education level. For this reason, there was no significant difference between the two groups regarding sexual disorders with the level of education. This finding does not mean that the level of education does not play a role in reducing sexual disorders in people. Still, it means that earthquake victims' educational level distribution is normal. Therefore, the knowledge level of earthquake victims does not affect increasing or decreasing the severity of sexual dysfunctions.

There was a statistically significant difference between the mean scores of IIEF and the level of social support from men affected by the earthquake. Sexual dysfunctions were more severe in men their families and friends did not support. Social support seems to reduce the severity of sexual dysfunction in earthquake‐affected people by lowering posttraumatic stress disorders. Warner et al.^35^ reported that receiving social support from relatives shortly after an earthquake was an important coping resource, as it alleviated the association between resource loss and the severity of posttraumatic stress response. Alipour and Ahmadi,^36^ In a systematic review, showed the positive effect of social support on the prevention of PTSD. Eray et al.^37^ showed that perceived family support has a protective role on PTSD symptoms of adolescents after the earthquake. In the current study, participants who received less social support were people who were unable to communicate with others and express their problems. Therefore, in disasters, people who are less likely to seek services should be given more attention in terms of mental disorders and especially sexual dysfunctions.

There was a statistically significant difference between the mean scores of IIEF and the presence or absence of underlying disease between the with and without sexual dysfunction groups. Venggadasamy et al.^38^ in a study showed that in men with diabetes, several issues might occur in sexual dysfunction, such as erectile dysfunction, orgasmic disorders, ejaculatory disorders, and reduced libido, and men with diabetes‐induced SDs experienced a poor quality of life due to the distress caused by SD. Terentes‐Printzios et al.^39^ a study showed an interaction between erectile dysfunction, cardiovascular disease, and cardiovascular drugs, and ED could be used as a predictor of vascular disorders. Therefore, since there was a positive relationship between ED and underlying diseases in the with and without sexual dysfunction groups, men with underlying diseases must be examined for sexual dysfunctions after the earthquake.

Sexual disorders were more severe in men not supported by their families and those not using psychological counseling services. There was no significant relationship between the use of psychological counseling after the earthquake between the groups with and without sexual disorders, and only 4% in the group with sexual disorders and 2.4% in the without sexual disorders group had used these services. Many studies have shown that the prevalence of psychosexual disorders increases after disasters. Also, the findings showed that people who had used psychological services had very low IIEF scores and were probably forced to use psychological services due to severe mental disorders. Men who used psychological services seemed exposed to postearthquake stress, anxiety, and depression. Sexual dysfunctions of these individuals may have been due to their mental disorders and using antidepressant and anxiety drugs. Yang et al.^40^ reported a significant association between anxiety and depression with sexual disorders. Only a minority of older adults seek help from healthcare professionals or use medication for SD.^26^

85.2% of the participants in both groups faced financial damages due to the earthquake. However, there was no significant association between financial damages and sexual dysfunctions. It seems that the reason for the insignificancy of this association is the high percentage of people affected by the earthquake. In contrast, Dadomo et al.^41^ reported a significant association between financial damages and erectile dysfunction.

Men injured due to the earthquake and those with at least one underlying disease (e.g., diabetes, asthma, heart problems, etc.) had more severe sexual dysfunction. In line with the current study's findings, Bahar et al.^42^ reported a significant association between diabetes and sexual dysfunction. Soto et al.^43^ also reported a significant association between asthma and the quality of sexual activity.

There was no significant association between having an adequate sleep in earthquake‐affected men and the severity of sexual dysfunction in both groups. Since most participants had a good or relatively good sleep, this finding was not significant in the present study. Consistent with the current study's findings, Pastuszak et al.^44^ reported a significant association between sleep quality and sexual disorders.

There was no significant association between adequate food intake and consumption of sufficient fruits and sexual dysfunction in both groups. Mykoniatis et al.^28^ showed in a study that greater consumption of vegetables and fruits, a lower intake of dairy and alcoholic beverages, and a less intense smoking habit increase sexual disorders in men.

There was a significant association between the availability of life facilities such as sanitary toilets and bathrooms between the groups with and without sexual dysfunctions. The authors did not find a study on the association between life facilities of earthquake victims and sexual disorders. However, it seems that the higher the living facilities of earthquake‐affected victims, the higher their physician and mental health would be, and they will have more desire for sexual activities.

In this study, a self‐report questionnaire was used to investigate sexual dysfunctions, and the researchers were not present when the participants completed the questionnaire. Some participants may not have answered some questions correctly, so this study is limited in this respect.

## 5 CONCLUSION

The prevalence of sexual dysfunction in men increased after the earthquake. Approximately half of the earthquake‐affected men had sexual dysfunctions. The men's age, child numbers, current live location, social support, underlying disease, and availability of sanitary toilets and bathrooms were influential in the severity of men's sexual dysfunctions after the earthquake. Therefore, crisis managers, policymakers, psychiatrists, and psychologists should pay enough attention to men's sexual dysfunction after earthquakes. This study was one of the first studies conducted on the status of sexual disorders in men after the earthquake. Therefore, it is suggested to conduct more studies in this field in other settings and populations. Also, it is suggested to conduct studies with the same design and examine the sexual disorders of earthquake victims 2–3 years after the earthquake because these disorders may improve or become more severe over time.

## AUTHOR CONTRIBUTIONS

**Abbasali Ebrahimian**: Conceptualization; formal analysis; methodology; project administration; resources; supervision; validation; writing – original draft; writing – review & editing. **Hossein Babaei**: Conceptualization; investigation; methodology; writing – original draft; writing – review & editing. All authors have read and approved the final version of the manuscript.

## CONFLICT OF INTEREST

The authors declare no conflict of interest.

## TRANSPARENCY STATEMENT

The lead author Hossein Babaei affirms that this manuscript is an honest, accurate, and transparent account of the study being reported; that no important aspects of the study have been omitted; and that any discrepancies from the study as planned (and, if relevant, registered) have been explained.

## Data Availability

The data that support the findings of this study are available on request from the corresponding author. The lead author Dr. Abbasali Ebrahimian has full access to all of the data in this study and takes complete responsibility for the integrity of the data and the accuracy of the data analysis.
